# Atrial Fibrillation Ablation with Multipolar Phased-Radiofrequency Catheter: The Learning Curve Effect for Procedural Parameters, But not for the Long-Term Outcome

**DOI:** 10.3390/jcm8101589

**Published:** 2019-10-02

**Authors:** Andrzej Glowniak, Adam Tarkowski, Katarzyna Wojewoda, Katarzyna Wysokinska, Mariusz Kozak, Piotr Wacinski, Andrzej Wysokinski

**Affiliations:** Department of Cardiology, Medical University of Lublin, 8 Jaczewskiego Str., 20-090 Lublin, Poland

**Keywords:** atrial fibrillation, pulmonary vein isolation, learning curve, blanking period, phased radiofrequency ablation

## Abstract

Background: Pulmonary vein isolation (PVI) is a routine treatment in atrial fibrillation (AF). Single-shot techniques were introduced to simplify the procedure. We analyzed time-dependent changes in procedural parameters, acute success, complication rates, and long-term outcomes during our initial experience with multipolar phased-radiofrequency (RF) ablation. Methods and Results: The first 126 consecutive patients (98 male; age: 58.8 ± 8.7 years) who underwent PVI with phased-RF ablation at our center were included in the study. Procedural parameters, complication rate, acute success and 12-month efficacy were compared in the first, second and third group of 42 consecutive patients. In all patients, 516/526 PVs were effectively isolated (98.1%), with no differences between the tierces (*p* = 0.67). Procedure (169.8 vs. 132.9 vs. 105.8 min, *p* < 0.0001), fluoroscopy (32.9 vs. 24.3 vs. 14.1 min, *p* < 0.0001) and left atrial dwell (83.0 vs. 61.9 vs. 51.4 min, *p* < 0.0001) times were significantly reduced with experience in tierces 1–3, respectively. In the 12-month follow-up, 60.3% of patients were arrhythmia-free with no differences between the tierces (*p* = 0.88). In multivariate analysis, the relapse in the blanking period (*p* < 0.0001), time from AF diagnosis (*p* = 0.004) and left atrial diameter (*p* = 0.012) were the only independent predictors of AF recurrence. Conclusions: The learning curve effect was demonstrated in procedural parameters, but not in the complication rate nor the long-term success of PVI with phased-RF technique. The relapse in the blanking period was the strongest predictor of treatment failure in long-time observation.

## 1. Introduction

Catheter ablation of atrial fibrillation (AF) has become a regular treatment option in patients with symptomatic recurrent episodes, refractory to antiarrhythmic drugs [1,2,3]. It is generally agreed that pulmonary vein isolation (PVI) is the cornerstone of the AF ablation [4]. The standard approach, with point-by-point circumferential isolation of pulmonary vein (PV) antra with irrigated radiofrequency (RF) catheter, is a time-consuming technique. To simplify and speed-up AF ablation procedure, new “single-shot” devices were developed [5,6,7,8], offering a way to isolate one vein in virtually “one-shot”. While a single shot is the goal, these devices often require several applications per vein. Nevertheless, they lead to important reduction of the procedure time, with the safety profile and long-term outcome similar to point-by-point isolation [9,10,11,12]. Single-shot techniques are faster to learn; however, as with all novel methods, it takes time even for experienced operators to adapt to the new technology. The learning curve effect for procedural parameters, similarly to procedure and fluoroscopy time, was demonstrated for two of the most commonly used single-shot devices: cryoballoon [13] and phased-RF circular ablation catheter [14]. Interestingly, the learning curve effect, reported for procedural parameters, was not described for long-term outcome except for one report regarding cryoballoon ablation [15]; however, it was linked with the shift in patients’ selection over time.

In this study, we analyzed time-dependent changes in procedural parameters, acute success, complication rate, and long-term outcome throughout our early experience with multipolar phased-radiofrequency (RF) catheter ablation. Additionally, we aimed to identify predictors of long-lasting arrhythmia-free survival.

## 2. Experimental Section

### 2.1. Study Population

A total of 126 consecutive patients (98 male; age: 58.8 ± 8.7 years) who underwent pulmonary vein isolation (PVI) with a multipolar circular phased-radiofrequency (RF) catheter at our center were included in the study. Exclusion criteria involved previous atrial fibrillation (AF) ablation, intracardiac thrombus, LA diameter > 50 mm, long-lasting persistent AF, thyroid dysfunction, left ventricular (LV) ejection fraction ≤ 40%, considerable valvular heart disease, heart failure of NYHA class III or IV, history of unstable angina/myocardial infarction within the past 6 months, contraindication to oral anticoagulants and pregnancy. Procedural parameters, acute success and 12-month efficacy were compared in the first, second and third group of 42 consecutive patients (tierce 1, 2 and 3). All analyzed patients were assigned to the tierces in a consecutive manner over the 51-month time span. The duration of the tierces 1, 2 and 3 was 20, 13 and 18 months (*p* = 0.317), respectively. The phased-RF procedures analyzed in this study accounted for 37.8%, 42.4% and 29.4% (*p* = 0.096) of the total number of the AF ablation procedures performed in our EP lab in the time-span covering the tierces 1, 2 and 3, respectively. Patient characteristics are presented in Table 1. The study protocol conforms to the ethical guidelines of the 1975 Declaration of Helsinki; this was approved by the institutional review board and written informed consent was given by all participants.

### 2.2. Periprocedural Management

A computed tomography (CT) scan or contrast angiography was performed to assess the anatomy of the left atrium and pulmonary veins. An anatomical variant of the left-sided common PV ostium was identified in 23/126 (18.3%) and additional right-sided pulmonary vein in 10/126 (7.9%) of the patients who were also eligible for the PVI procedure. All patients were administered oral anticoagulation prior to the procedure. With vitamin K antagonists (VKAs), an uninterrupted anticoagulation strategy was used, targeting the therapeutic international normalized ratio (INR) value [16]. In the case of non-vitamin K oral anticoagulants (NOACs), we accepted an interrupted schedule, with the preceding dose administered 12 h (dabigatran/apixaban) to 24 h (rivaroxaban) before and the next dose 6 h after the ablation. In all patients, pre-procedural transesophageal echocardiography (TEE) was performed to rule out left atrial thrombus.

### 2.3. Ablation Procedure

The pulmonary vein isolation technique with circular multipolar catheter (PVAC, Medtronic, Minneapolis, MN, USA) and duty-cycled phased-radiofrequency energy generator (Genius, Medtronic, Minneapolis, MN, USA) was described in detail elsewhere [6,7,8,17]. Both operators involved in the study had previous experience with point-by-point AF ablation. The majority of the procedures were performed by A.G., with no significant differences between the groups: 38/42 (90.5%), 35/42 (83.3%) and 34/42 (81%) in tierces 1, 2 and 3, respectively (*p* = 0.44). Procedures were performed with local anesthesia under conscious sedation. After two punctures in the right femoral vein, a steerable quadripolar diagnostic catheter was introduced into the coronary sinus (CS). A transseptal puncture was completed under fluoroscopy with a 10F (SL0, St. Jude Medical, St. Paul, MN, USA) sheath, constantly flushed with heparinized saline (1 unit/mL) throughout the procedure. A loading dose of 5.000 I.U. heparin was administered before the transseptal puncture, with the following dose given directly after crossing the septum to achieve a total dose of 70 IU/kg; then, the activated clotting time (ACT) was checked at 20-minute intervals, with additional heparin administration if required, to maintain a target ACT of 300 s or above. All ablations were performed with a circular multipolar pulmonary vein ablation catheter (PVAC/PVAC Gold, Medtronic, Minneapolis, MN, USA) together with a dedicated multi-channel phased-RF generator (Genius, Medtronic, Minneapolis, MN, USA). A PVAC Gold catheter was used in the last ten cases. To reduce the risk of embolic events, the submerged PVAC catheter loading technique was applied in all cases, even before the ERACE trial recommendations, confirmed by the following reports [18,19,20]. In all cases, the PVAC catheter was used for both mapping and ablation. After inserting a dedicated guidewire (PV-Tracker, Medtronic, Minneapolis, MN, USA) into a pulmonary vein, the PVAC catheter was delivered over-the-wire and placed against the PV ostium. Before the applications in the right-sided veins, pacing from each pair of electrodes was performed to determine area s of phrenic nerve capture. After antral signals were clearly identified, the phased radiofrequency energy applications—lasting 60 s each—were delivered with a unipolar to bipolar ratio of either 4:1 (8 W) or 2:1 (10 W), until PV isolation was completed. In the case of remaining single gaps, adequate pairs of electrodes were used for selective applications, with all others cut-off (an equivalent of routinely used touch-up applications with a single-tip catheter). Subsequently, isolation of all pulmonary veins was carefully evaluated, yet without the adenosine/isoproterenol challenge. In the case of early reconnection, the re-isolation was performed before the termination of the procedure. Immediately after the procedure and on the following day, a transthoracic echocardiogram (TTE) was performed to rule out pericardial effusion.

### 2.4. Follow-Up

The follow-up visits were scheduled at 1, 3, 6, 9, and 12 months after hospital discharge for all patients. At all follow-up visits, a 12-lead electrocardiogram (ECG) was recorded. At 3- and 6-month visits, a 24 h Holter monitoring was obtained, whilst a 72 h Holter was recorded at the 12-month follow-up. All Holter and ECG recordings completed in referring centers were sent to the Department of Cardiology, Medical University of Lublin for confirmation. Furthermore, symptom-driven ECG records and provisional visits were encouraged, including the 3-month post-procedural blanking period (BP). All recognized episodes of any atrial tachyarrhythmia beyond the BP lasting 30 s or more were considered as a recurrence. Atrial tachyarrhythmia episodes in the first 3 months after the procedure were considered as a relapse in the blanking period. Oral anticoagulation was continued for 3 months; afterwards, the decision regarding long-term anticoagulation was based on the CHA_2_DS_2_-VASc score. Post-procedural antiarrhythmic medication was continued for at least 3 months.

### 2.5. Statistical Analysis

Statistical analysis was performed with suitable statistical tests and Statistica software (Stat Soft, Tulsa, OK, USA). The values were presented as means and standard deviations (SD), medians, minimum and maximum value, and the range of variability. Normal distribution of continuous variables was tested with a Shapiro–Wilk test. The Student’s t-test or Mann–Whitney U-test for independent variables was used for intergroup comparison. The distribution of discrete variables in groups was compared with Pearson’s Chi-square test or Fisher’s exact test. Predictors of AF recurrence were analyzed by univariate and subsequent multivariate Cox regression analyses. The survival curves were constructed with the Kaplan–Meier method and the proportions of survivors within the groups were compared with the log-rank test. The multivariate prediction models for time to recurrence after the final ablation procedure were applied using stepwise regression based on likelihood ratios. The results were expressed as hazard ratios with 95% confidence intervals (CIs). The error was set at 5% and *p*-value < 0.05 was considered significant.

## 3. Results

### 3.1. Procedural Parameters and Acute Effects of Pulmonary Vein Isolation

**Figure 1:**
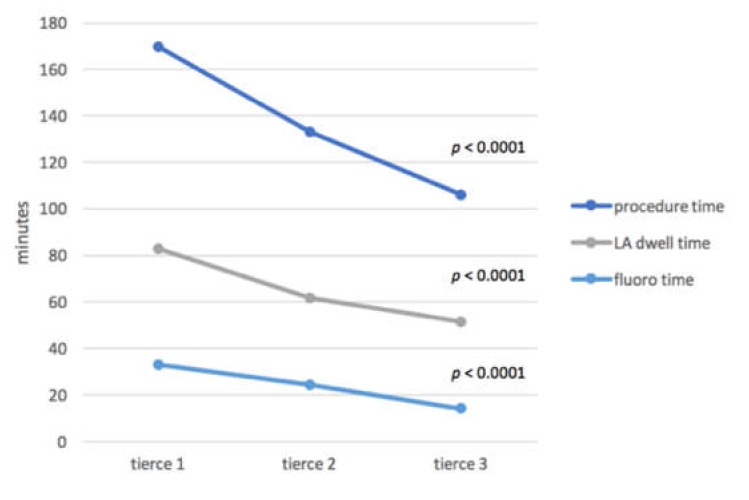
Procedure, left atrial (LA) dwell and fluoroscopy times in each tierce.

In 126 patients, a total of 526 pulmonary veins were identified by CT scan or contrast venography. Out of this number, 516 PVs (98.1%) were successfully isolated with the PVAC catheter, with no differences between the tierces 1, 2 and 3 (97.7% vs. 98.9% vs 97.8%, *p* = 0.67). In the majority of patients (93/126, 73.8%), focal RF applications with selected pairs of PVAC electrodes were used in the case of remaining gaps as an equivalent to touch-up applications with a single-tip catheter. The additional focal applications were necessary in 33 out of 42 patients (78.6%) within the 20-month-lasting tierce 1, in 28 out of 42 patients (66.7%) within the 13-month-lasting tierce 2 and in 31 out of 42 patients (76.2%) within the 18-month-lasting tierce 3. There were no significant differences in the number of focal applications between the three tierces (*p* = 0.422). The left superior pulmonary vein (LSPV) was most frequently targeted in all tierces. The LSPV was aimed at 29 out of 33 patients requiring additional applications in tierce 1 (87.9%), in 24 out of 28 patients requiring additional applications in tierce 2 (85.7%) and in 27 out of 32 patients requiring additional applications in tierce 3 (84.4%). There were no significant differences regarding the rate of LSPV applications between the three tierces (*p* = 0.919). In 10 out of 526 PVs (1.9%), complete isolation was not achieved due to the anatomical course of the phrenic nerve across the potential ablation site. There was significant shortening of the procedure time (169.8 min vs.132.9 min vs. 105.8 min, *p* < 0.0001), fluoroscopy time (32.9 min vs. 24.3 min vs. 14.1 min, *p* < 0.0001), LA dwell time (83.0 min vs. 61.9 min vs. 51.4 min, *p* < 0.0001) and total number of RF applications per procedure (24 (24–30) vs. 24 (20–28) vs. 23 (20–26), *p* < 0.0024) in tierces 1–3, respectively (Figure 1).

The only major complication was cardiac tamponade managed with pericardiocentesis in one patient (0.7%) in tierce 2. Minor complications involved limited groin hematoma in eight patients (6.3%), all of them were resolved without any additional treatment. There was no significant difference in complication incidence between the tierces (*p* = 0.32). The summary of procedural parameters, complications and results of pulmonary vein isolation is presented in Table 2.

### 3.2. Relapse in the Blanking Period

During the first 3 months after the ablation procedure, 66 out of 126 patients (52.4%) had documented AF episodes, considered as a relapse in the blanking period. There were no differences between tierces 1, 2 and 3 regarding relapse incidence (57.1% vs. 45.2% vs. 54.8%, *p* = 0.51). 

### 3.3. Long-Term Follow-up

**Figure 2:**
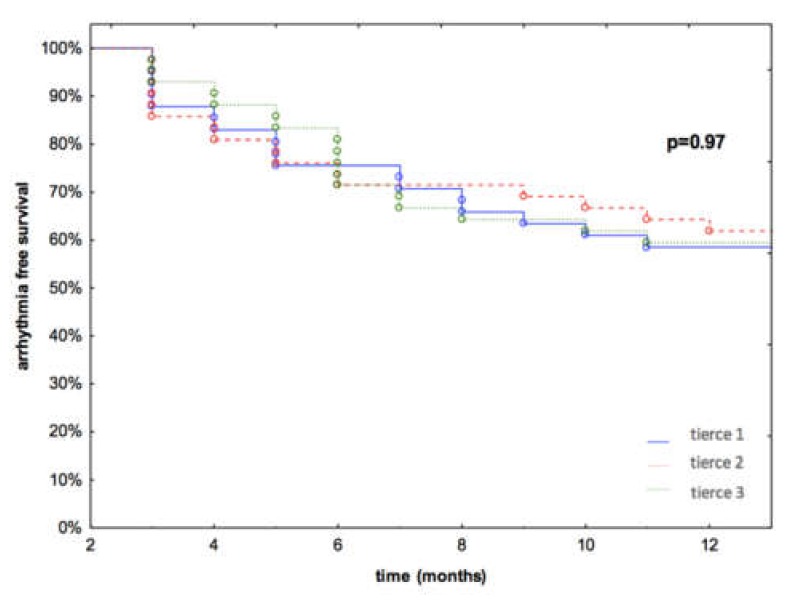
Arrhythmia-free survival in each tierce.

Atrial arrhythmia-free survival rates at 12 months after the procedure were 57.1%, 61.9% and 61.9% in tierce 1, tierce 2 and tierce 3, respectively (*p* = 0.88) (Figure 2). In the 12-month follow-up, 8 patients (2, 3 and 3 in tierce 1, 2 and 3, respectively) underwent repeated AF ablation procedures due to symptomatic recurrences of atrial fibrillation. In all of them, at least one pulmonary vein presented electrical reconnection, with the left superior PV being most frequently affected (2/2, 3/3 and 2/3 in tierce 1, 2 and 3, respectively). In multivariate Cox regression analysis, the time from AF diagnosis (*p* = 0.003), LA diameter (*p* = 0.012), and relapse in the blanking period (*p* < 0.0001) were the only independent predictors of AF recurrence in the long-term follow-up (Table 3).

## 4. Discussion

In our group, we managed to effectively isolate 98.1% of all pulmonary veins in 118 out of 126 patients (93.7%), with a low complication rate and acceptable arrhythmia-free survival (60,3%) at the 12-month follow up. These results are comparable with previously published data, regarding the phased-radiofrequency technique [6,7,8,9,10,21], including the recently published initial results from the multicenter GOLD AF registry [22], in which complete acute PV isolation was achieved in 93.8% of patients. Interestingly, the relapse in the blanking period was the strongest independent predictor (*p* < 0.0001) of AF recurrence in long-term follow-up, which stands in line with earlier observations regarding both RF [23,24,25] and cryoablation [26] and supports the opinion that blanking-period relapses are possible signs of early PV reconnection.

During the ablation procedure, in the majority of patients (73.8%), additional “focal” applications with selected pairs of electrodes were performed in order to achieve bidirectional PV isolation. There were no differences in proportions of additional applications between the tierces. Interestingly, the left superior pulmonary vein was the most frequent target for the additional applications in all tierces, both during the index ablation and during the repeated procedures, performed in eight patients during the 12-month follow-up period. The LSPV being most prone to both early (intraprocedural) and late reconnections is an interesting finding, as reported previously [26]. The possible explanation is the crucial role of the ridge between LSPV and left atrial appendage as a common reconnection site.

Furthermore, we have demonstrated the learning curve effect in pulmonary vein isolation with a PVAC catheter for procedural parameters: procedural and fluoroscopy times, left atrial dwell time and number of RF applications were significantly reduced with growing experience. However, we did not observe this effect for acute success, rate of complications and middle (relapse in the 3-month blanking period), as well as long-term arrhythmia-free survival. There is limited data regarding the learning curve effect in atrial fibrillation ablation. In the early report by Spragg et al. [27], who analyzed a large cohort of consecutive patients undergoing single-tip point-by-point pulmonary vein isolation, complication rates were higher during the first 100 (9.0%) than during the subsequent 541 (4.3%) cases. Sairaku et al. [28], who likewise analyzed data regarding point-by-point PVI, demonstrated that the learning curve effect for procedural time and complication rate both reduced with time, in addition to 6-month arrhythmia-free survival improving with growing experience. Similarly, the learning curve effect was demonstrated for single-shot PVI techniques. With both cryoballoon [13] and phased RF [14] ablation, a significant shortening of the procedure time was demonstrated, with no substantial change in the effectiveness over time. In one observational study regarding cryoballoon PVI, gradual improvement of the procedural outcome was reported; however, it was linked with a more careful selection of patients [15]. 

In our study, we have demonstrated the learning curve effect for procedural parameters; however, not for the acute success, complication rate and long-term outcome. Keeping in mind all of the differences between the two techniques, phased-RF pulmonary vein isolation seems to have a steeper learning curve compared to classical point-by-point isolation [28]. The main difference is the lack of learning curve effect regarding complication rate and short and long-term procedural outcome in the phased-RF technique. In other words, this technology can be quickly and safely adopted by operators with previous experience with transseptal access and atrial fibrillation ablation, with the prospect of good clinical outcome from the starting point.

### Study Limitations

There are several limitations of our study. Firstly, it is a single-center, observational study performed on a relatively small number of patients; therefore, with limited statistical power. Secondly, the operators were experienced with both classical point-by-point and cryoballoon PVI techniques, thus the demonstrated quick adoption to the phased-RF technique might not apply to electrophysiologists with limited experience with AF ablation. Furthermore, the relatively small representation of patients with atypical PV anatomy in tierce 1 and moderately older patients in tierce 3 could possibly influence the results, flattening the learning curve. Additionally, in the last ten cases, a modified PVAC Gold catheter was used, which might have influenced procedural parameters; yet, this would not affect the results in the first and second tierces.

## 5. Conclusions

We have analyzed time-dependent changes in procedural parameters, complication incidence, acute success and long-term outcome during the initial experience with a phased-RF multipolar ablation catheter. The steep learning curve effect was demonstrated for procedural and LA dwell times and total number of RF applications; however, not on the complication rate, acute success and 12-month arrhythmia-free survival. The relapse in the blanking period was the strongest predictor of treatment failure in long-time observation.

## Figures and Tables

**Table 1:** Baseline characteristics of the patients.

Characteristics	Tierce 1 (*n* = 42)	Tierce 2 (*n* = 42)	Tierce 3 (*n* = 42)	*p* Value
age in years, mean ± SD	57.4 ± 9.1	57.6 ± 8.1	61.7 ± 8.4	0.034
male gender, n (%)	32 (32.7)	32 (32.7)	34 (34.7)	0.83
BMI in kg/m^2^, mean ± SD	27.7 ± 3.1	27.8 ± 2.7	26.7 ± 2.7	0.21
congestive heart failure, n (%)	2 (4.8)	3 (7.1)	0 (0)	0.23
hypertension, n (%)	31 (73.8)	25 (59.5)	31 (73.8)	0.26
diabetes, n (%)	6 (14.3)	8 (19.1)	7 (16.7)	0.84
previous stroke, n (%)	0 (0)	1 (2.4)	0 (0)	0.36
vascular disease, n (%)	5 (11.9)	7 (16.7)	9 (21.4)	0.50
CHA2DS2-VASc score, mean ± SD	1.5 ± 1.1/1 (0–5)	1.5 ± 1.3/1.5 (0–5)	1.7 ± 1.0/1.5 (0–4)	0.69
LA diameter in mm, mean ± SD	43.0 ± 2.7	43.0 ± 3.6	42.7 ± 3.0	0.85
LVEF (%), mean ± SD	60.2±5.2	59.4±6.1	60.0±5.1	0.75
atypical PV anatomy, n (%)	7 (16.7)	18 (42.9)	16 (38.1)	0.024
paroxysmal AF, n (%)	40 (95.2)	39 (92.9)	39 (92.9)	0.88
time from diagnosis, median (range)	41 (12–132)	32.5 (12–144)	30 (12–144)	0.32

Abbreviations: BMI—body mass index; LA—left atrium; LVEF—left ventricular ejection fraction; PV—pulmonary vein; AF—atrial fibrillation; SD—standard deviations.

**Table 2:** Procedural characteristics, complications and follow-up.

Characteristics	Tierce 1 (*n* = 42)	Tierce 2 (*n* = 42)	Tierce 3 (*n* = 42)	*p* value
procedure time, mean ± SD	169.8 ± 27.5	132.9 ± 20.3	105.8 ± 19.7	<0.0001
LA dwell time, mean ± SD	83.0 ± 18.6	61.9 ± 5.5	51.4 ± 7.5	<0.0001
fluoroscopy time, mean ± SD	32.9±7.7	24.3±5.9	14.1±4.9	<0.0001
No of RF applications, median (range)	24 (24–30)	24 (20–28)	23 (20–26)	0.0024
complications, n (%)	3 (7.1)	3 (7.1)	3 (7.1)	0.32
acute effect (no of veins isolated), (%)	169/173 (97.7)	172/174 (98.9)	175/179 (97.8)	0.67
relapse in the blanking period, n (%)	24/42 (57.1)	19/42 (45.2)	23/42 (54.8)	0.51
arrhythmia free after 12 months, n (%)	24 (57.1)	26 (61.9)	26 (61.9)	0.88

Abbreviations: LA—left atrium; RF—radiofrequency.

**Table 3:** Uni- and multivariate Cox proportional hazard ratios for AF recurrence in 12-month follow-up.

Parameter	Univariate		Multivariate	
	HR (95% CI)	*p* Value	HR (95% CI)	*p* Value
age	1.04 (0.99–1.08)	0.13		
sex	0.89 (0.40–2.01)	0.82		
AF type	2.09 (0.71–6.17)	0.18		
EHRA	1.51 (0.84–2.74)	0.17		
procedure time	0.99 (0.98–1.01)	0.48		
LA dwell time	1.01 (0.98–1.04)	0.42		
CHA_2_DS_2_-VASc	0.88 (0.59–1.32)	0.53		
body mass index	1.00 (0.90–1.11)	0.99		
LA diameter	1.15 (1.02–1.30)	0.027	1.14 (1.03–1.26)	0.012
LV ejection fraction	0.98 (0.92–1.04)	0.58		
atypical PV anatomy	0.70 (0.34–1.44)	0.33		
time from AF diagnosis	1.01 (1.00–1.02)	0.0044	1.01 (1.00–1.02)	0.003
relapse in blanking period	7.34 (3.19–16.89)	<0.0001	6.67 (3.02–14.74)	<0.0001

Abbreviations: AF, atrial fibrillation; LA, left atrium; LV, left ventricle; PV, pulmonary vein; CI, confidence intervals; EHRA, European Heart Rhythm Association score.
